# Genome survey and high-density genetic map construction provide genomic and genetic resources for the Pacific White Shrimp *Litopenaeus vannamei*

**DOI:** 10.1038/srep15612

**Published:** 2015-10-27

**Authors:** Yang Yu, Xiaojun Zhang, Jianbo Yuan, Fuhua Li, Xiaohan Chen, Yongzhen Zhao, Long Huang, Hongkun Zheng, Jianhai Xiang

**Affiliations:** 1 Key Laboratory of Experimental Marine Biology, Institute of Oceanology, Chinese Academy of Sciences, Qingdao 266071, China; 2 Guangxi Key Laboratory of Aquatic Genetic Breeding and Healthy Aquaculture, Guangxi Academy of Fishery Sciences, Nanning 530021, China; 3 Biomarker Technologies Corporation, Beijing 101300, China

## Abstract

The Pacific white shrimp *Litopenaeus vannamei* is the dominant crustacean species in global seafood mariculture. Understanding the genome and genetic architecture is useful for deciphering complex traits and accelerating the breeding program in shrimp. In this study, a genome survey was conducted and a high-density linkage map was constructed using a next-generation sequencing approach. The genome survey was used to identify preliminary genome characteristics and to generate a rough reference for linkage map construction. De novo SNP discovery resulted in 25,140 polymorphic markers. A total of 6,359 high-quality markers were selected for linkage map construction based on marker coverage among individuals and read depths. For the linkage map, a total of 6,146 markers spanning 4,271.43 cM were mapped to 44 sex-averaged linkage groups, with an average marker distance of 0.7 cM. An integration analysis linked 5,885 genome scaffolds and 1,504 BAC clones to the linkage map. Based on the high-density linkage map, several QTLs for body weight and body length were detected. This high-density genetic linkage map reveals basic genomic architecture and will be useful for comparative genomics research, genome assembly and genetic improvement of *L. vannamei* and other penaeid shrimp species.

The Pacific white shrimp *Litopenaeus vannamei* is native to the eastern Pacific Ocean. It was first introduced into China in the late 1980s. By 2010, it had become the major cultured shrimp species, accounting for 85% of the total shrimp production in China. It is also extensively cultivated in southern Asia and northern and southern Africa (FAO Fishery Statistics, 2006). In 2012, the total world production of *L. vannamei* was 3,178,721 tons, making this species the dominant crustacean species in global seafood mariculture. During the past decade, large efforts have been made to investigate the genome and genetic architecture of this species, including BAC library construction^1^, BAC end sequencing^2^, transcriptome sequencing^3^,^4^,^5^, molecular marker development^6^,^7^,^8^,^9^,^10^,^11^, and linkage map construction^12^,^13^. To comprehensively understand the genomic and genetic characteristics of this species, whole genome sequencing and a high-density linkage map are necessary. However, the *L. vannamei* genome is large and contains highly repetitive sequences^1^,^2^, which present significant challenges for the whole genome sequencing project and other genetic studies.

A robust, high-density genetic linkage map is a useful tool for genome assembly, as well as for mapping quantitative trait loci (QTL) of economically important traits^14^,^15^. Recently, genetic linkage maps have been constructed for many aquaculture species, such as rainbow trout^16^, Atlantic salmon^17^, catfish^18^, grass carp^19^, Kuruma prawn^20^, black tiger shrimp^21^, and others. Several linkage maps have been constructed for *L. vannamei*. The linkage map constructed using AFLP markers proved difficult to be used in different families, which limited usage for QTL mapping and gene cloning^12^,^22^,^23^. Although the SSR and gene-based SNP maps provided more genetic information for *L. vannamei*, the map density and coverage need to be increased^13^,^24^. Therefore, a high-density linkage map is necessary for genomic and genetic studies in shrimp species.

Next-generation sequencing (NGS) technology has extended our ability to conduct *de novo* genome sequencing and high density linkage construction for non-model species. One of the major methods used to construct high-density linkage maps, called restriction-site-associated DNA (RAD) sequencing, has been widely used in non-model species^25^. This method has also been applied in QTL mapping, population genetic studies and comparative genome studies^26^. Using RAD sequencing, high-density genetic linkage maps have been constructed for grapes^27^, gudgeons^28^, Atlantic halibut^29^, and other species. Recently, a similar method of *de novo* SNP discovery and genotyping, called specific-length amplified fragment sequencing (SLAF-seq), was reported^30^. Based on deep sequencing and double barcode genotyping systems, this method was accurate and cost-effective for linkage map construction. A density linkage map, including 5,885 markers, was constructed for common carp using this method, with marker intervals of 0.68 cM on average^30^. Moreover, high-density linkage maps for sesame, soybean, and cucumber have also been constructed using SLAF-seq^31^,^32^,^33^,^34^.

One of the main purposes of creating a high-density linkage map is to make the mapping of QTLs for various traits possible. Among the traits of interest in shrimp, body weight and body length are the most important because both have high commercial significance in aquaculture. To date, only one QTL mapping study for *L. vannamei* has been published based on AFLP and SSR markers; three QTLs for body weight and body length were identified^23^. As this linkage map was constructed primarily with AFLP markers and the marker interval was large (7.6 cM), further mapping and cloning of growth related genes would be difficult.

In this study, for the first time, we combined a genome survey analysis and construction of a high-density linkage map to investigate the genomic and genetic architecture of *L. vannamei*. Based on the high-density linkage map, QTL mapping was conducted to detect markers related to growth traits.

## Results

### Genome survey of *L. vannamei*

Three paired-end DNA libraries with insert sizes of 170 bp, 300 bp and 500 bp were constructed and sequenced for the genome survey analysis. A total of 138.77 Gb of sequencing data were generated. After filtering out the adapter sequences and low quality and duplicated reads, a total of 90.69 Gb high-quality reads were retained, covering approximately 37-fold genome size of *L. vannamei*. The calculated GC content was 38.16%. The frequencies of 17-mers (nucleotide strings with a length of 17 bp) among the raw sequencing data were calculated, and a K-mer curve was constructed (Supplementary Figure S1). K-mer analysis revealed that there was a peak at the K-mer depth of 22. Genome size, G, was estimated as 2.64 Gb according to the following empirical formula: G = K_num/K_depth, where K_num is the total number of K-mers, and K_depth is the maximal frequency^35^. Compared to the *L. vannamei* genome size of 2.45 Gb estimated by flow cytometry^36^, the estimate of 2.6 Gb is closer to the true value. Based on this result, a remarkably high percentage of repetitive sequences (~79.37%) was estimated in the *L. vannamei* genome.

*De novo* assembly of the *L. vannamei* genome was conducted using these sequencing data with SOAPdenovo software. Two additional mate-paired sequencing datasets (Table 1) were added for a total dataset of 108.85 Gb, which was 41.86-fold the size of the genome. As a result, a total of 6,908,022 contigs with an N50 size of 409 bp were produced (Table 2). The assembled contigs covered 2,306,928,471 bp of the genome. Scaffolds with N50 of 1.34 Kb were also generated, with the longest scaffold reaching 38 Kb. All the sequencing reads were realigned to the contigs with the help of SOAPaligner (http://soap.genomics.org.cn/soapaligner.html), and >93% of the sequenced reads remapped into contigs.

### SLAF-tag generation and marker genotyping

**Figure 1:**
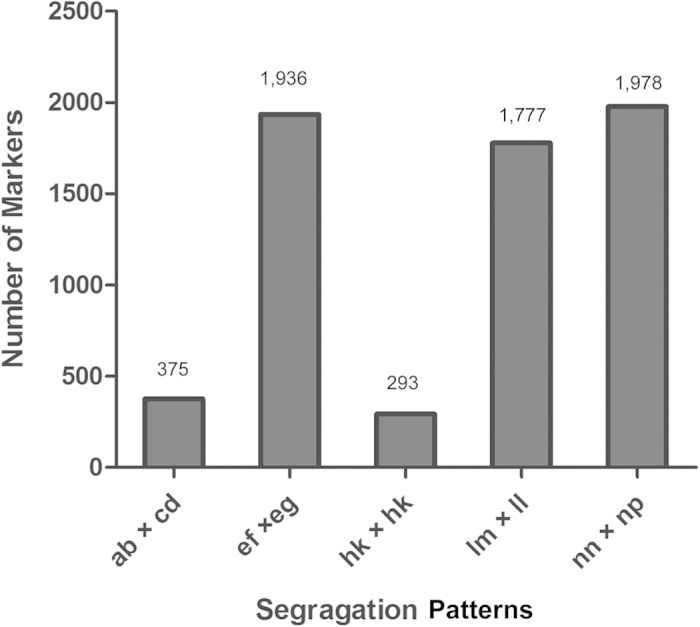
Statistics of genotyped SLAF markers in six segregation patterns.

SLAF sequencing of the mapping family with the Illumina HiSeq 2500 platform generated 456,620,260 paired-end reads for the parents and 205 progenies. The average number of total reads for parents and offspring were 10,233,561 and 2,127,576, respectively (Supplementary Table S1). A total of 114,829 SLAF markers were detected, of which 25,140 were polymorphic (Table 3). Among these 25,140 polymorphic markers, 6,359 were successfully genotyped in both parents and offspring. With a full-sib family design, SLAF markers could be classified into five segregation patterns (ab × cd, ef × eg, hk × hk, lm × ll, nn × np) (Fig. 1). Statistical analysis of the segregation patterns showed that nn × np was the major pattern, followed by ef × eg and lm × ll. The average read depth of genotyped markers ranged from 7.79 to 24.29 in the offspring. In the male and female parents, the average read depth was 61.47 and 55.07, respectively (Supplementary Table S2).

### Linkage mapping

A total of 6,359 high-quality markers were available for the linkage map construction using a pseudo-testcross strategy. The final linkage map contained 44 linkage groups, including 4,201 markers in the male map, 4,396 markers in the female map, and 6,146 markers in the sex-averaged map (Supplementary Data S1, Supplementary Table S3, Supplementary Figure S2–S6). The group LOD value ranged from 3 to 5 depending on the linkage group. The total map distances for the three maps were 6,143.95 cM (male map), 5,657.42 cM (female map), and 4,271.43 cM (sex-averaged map). The mean distance between two markers was 1.46 cM (male map), 1.29 cM (female map), and 0.7 cM (sex-averaged map) (Table 4).

### Marker distribution and intermarker distance

**Figure 2:**
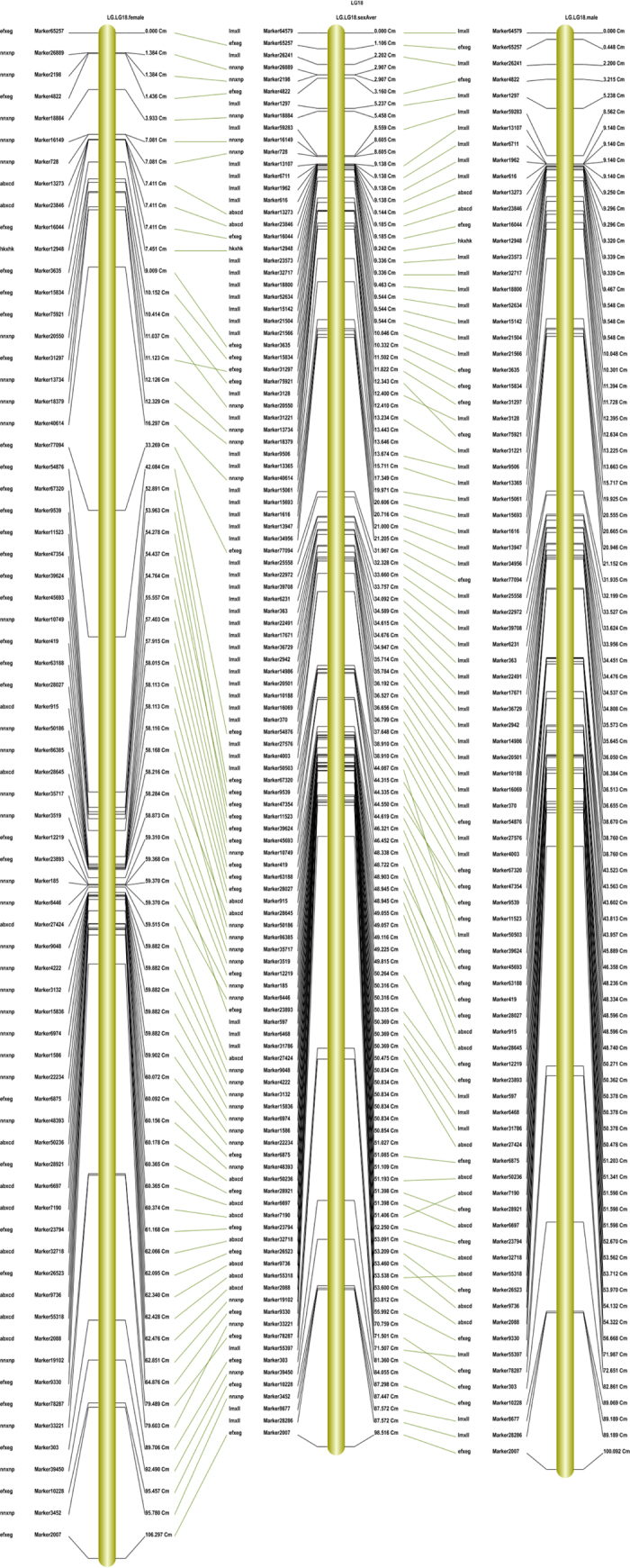
Demonstration of synteny analysis between female, male and sex average map.

The distribution of markers among linkage groups was uneven. In the male, female, and sex-averaged maps, the largest linkage group was Linkage group 1 (LG 1), which contained 182, 204, and 279 markers, respectively. The smallest linkage group was LG 14, which contained 16, 11 and 21 markers, respectively (Table 4). To evaluate the marker distribution, we analyzed the marker interval in each linkage group. On average, 91% of the female map, 93% of the male map, and 96% of the sex-averaged map were covered by markers with interval distances of less than 5 cM (Supplementary Table S4). A synteny analysis among the male map, female map, and sex-averaged map was also performed. The consistency of the marker distribution among the three maps was 90% (Fig. 2). The intermarker distance in the sex-averaged map ranged from 0 to 36.8 cM, with an average of 0.7 cM (Table 4). Most (84%) marker intervals were less than 1 cM, and 48 marker intervals were longer than 10 cM.

### Genome length and coverage estimation

The estimated total genome map length was 6,301.94 cM (male map), 5,790.27 cM (female map), and 4,341.39 cM (sex-averaged map). Based on this estimated total genome map length, the genome coverage of the male, female, and sex-average linkage map was 97.49%, 97.71% and 98.39%, respectively. With an estimated genome size of 2.6 Gb and linkage map length of 4,341.39 cM, the relationship between physical and genetic distances was estimated as 598.89 Kb/cM. Thus, the estimated physical distance between the adjacent markers of the sex-averaged map ranged from 0 Kb to 22.1 Mb, with an average of 419.22 Kb.

### Segregation distortion markers on the map

In total, 406 segregation distortion markers were mapped to the sex-averaged map (P < 0.05). These markers accounted for 6.61% of the total mapped markers (Table 5). Marker segregation with P values > 0.01 (chi-square test) represented 87.08% of the total mapped markers.

### Sex differences in recombination rates

The female and male genetic linkage maps exhibited different marker numbers and different recombination rates. In general, the female map contained more markers than the male map, but map distances were shorter. The method used to estimate the ratio of female to male recombination rate has been previously described^18^. Briefly, the common informative markers between the female and male maps were extracted. The map length for each linkage group containing the common markers was calculated separately. Sex differences were represented by the female map length divided by male marker length between the common markers. As a result, a total of 2451 markers were the same between the female and male maps. Based on these common markers, the male map was 5,885.74 cM and the female map was 5,578.20 cM (Table 6). The ratio of female/male recombination rates was 1/1.06 over all the linkage groups; however, the ratio differed between groups. The ratio ranged from 0.23 to 3.21 among the 44 linkage groups. In 19 of 44 linkage groups, a higher recombination rate was observed in male maps. In the other 25 linkage groups, higher recombination rates were observed in the female map.

### Linkage map integration

Of the 6,146 markers in the sex-averaged linkage map, 5,922 markers could be anchored to 5,885 scaffolds. Among these, 2,262 markers had high confidence, with both ends of the marker unambiguously anchored to the same scaffold.

**Figure 3:**
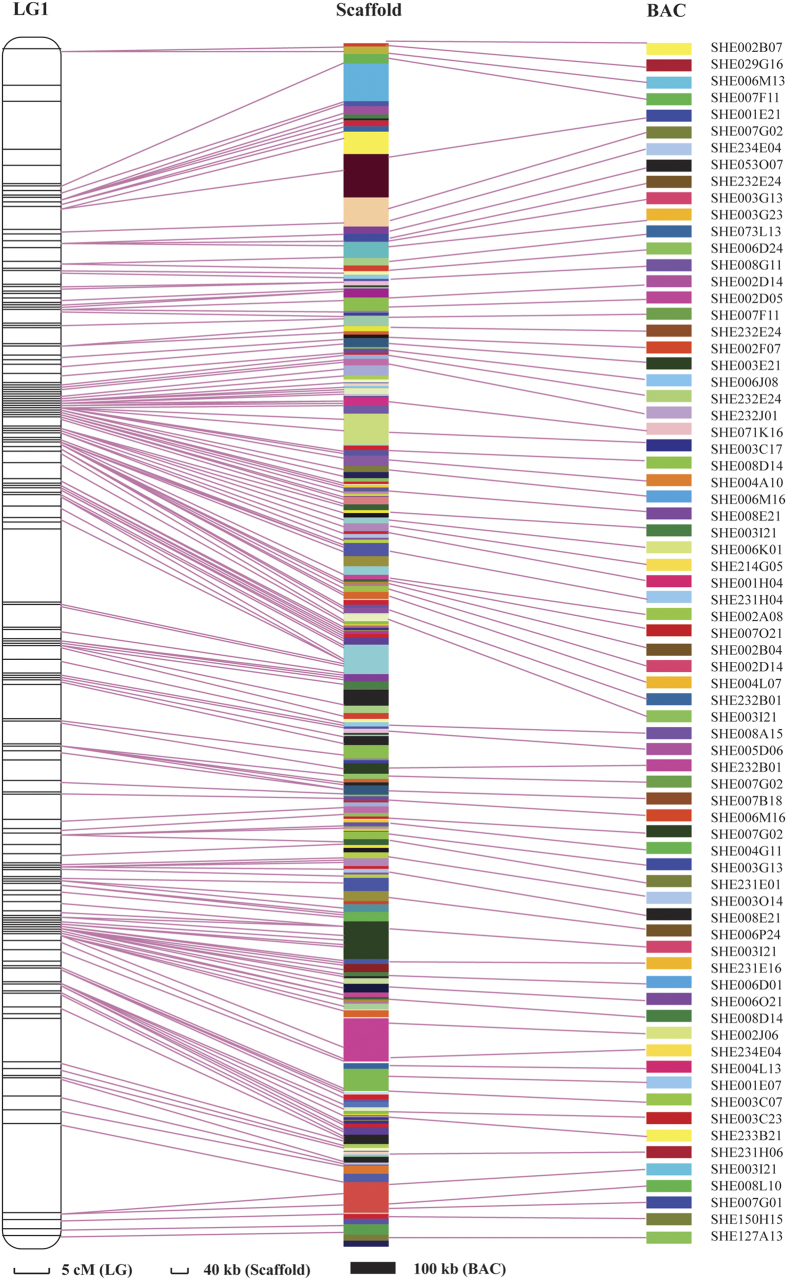
Demonstration of the integration of linkage group LG1, genomic scaffolds and BAC clones.

A BAC library is another important genomic resource. In a previous study, 11,279 BESs (BAC-end sequences), including 4,609 paired-end BESs were obtained by Sanger sequencing^2^. When comparing these BESs with the marker-anchored scaffolds, 1,504 BAC clones (302 BAC clones with both ends of the BESs matched to the same scaffold) were homologous to the scaffolds and could be linked with the linkage map. As a result, the information from the linkage maps, genomic scaffold and BAC clones could be integrated (Fig. 3).

### QTL mapping of growth traits

Both body weight and length followed a normal distribution. The estimated significant thresholds from permutation tests were 5.0 and 3.5 for body length and body weight, respectively. Using the Composite Interval Mapping method, a total of 11 significant QTLs for body length were detected (Table 7). The QTLs with the highest LOD score, LOD 6.5, were located at 25.9 cM of LG33 near Marker24250. The proportion of phenotypic variation explained by this QTL was 17.9%. The other QTLs were detected on nine different linkage groups. Except for two QTLs on linkage group 38, the other eight linkage groups contained only one QTL. The nearest markers for each QTL position are shown in Table 7. Using MIM analysis, the total genetic variance explained by all of the QTLs was estimated as 38.56%. For body weight, the predominant QTL was located at 46 cM on LG9, with an LOD score of 7.1 (Table 8). The nearest marker was Marker34000. The other QTLs were detected on LG10, LG22, LG27, LG35, LG38 and LG41, mapping to Marker7605, Marker33688, Marker21173, Marker58445, Marker4670 and Marker10074, respectively. The total genetic variance explained by all of these QTL was 17.65% from the result of MIM analysis.

## Discussion

The purpose of this study was to construct a high-density genetic linkage map for *L. vannamei* to assist with further genome assembly and QTL mapping studies. Previously, no genome reference was available. Therefore, we first conducted a genome survey analysis to describe the basic characteristics of the *L. vannamei* genome. Secondly, we used a SLAF-seq approach to construct a high-density linkage map with 6,146 markers spanning 44 linkage group.

Genome sequencing has been an important step for deciphering molecular mechanisms and accelerating genetic improvements of traits of interest in economically important species. However, although *L. vannamei* is one of the most important marine aquaculture species, few studies have investigated its genome. In a previous study, the BAC library of *L. vannamei* proved difficult to analyze, suggesting that shrimp DNA might have some unique characteristics^1^. Moreover, a high ratio of repetitive sequence and high heterozygosity were observed in the BAC-end sequence and transcriptomic SNP analyses^2^,^11^. In our study, the genome survey analysis highlighted the complexity of the *L. vannamei* genome. Approximately 80% of the genome was occupied by repetitive sequences, which was very similar to the *N. denticulate* genome^37^. The K-mer curve was quite different from those reported in other species^35^. This difference might be caused by the high ratio of repetitive sequences and other special characteristics of shrimp DNA. A primary reference genome was assembled based on the genome survey of sequencing data. The high ratio of repetitive sequences made the assembly procedure difficult, and relative short N50 contigs and scaffold sizes were obtained. Therefore, from the genome survey, we inferred that the assembly of the whole genome sequence may be greatly challenging if only using data generated by Illumina sequencing technology. Other sequencing methods such as the PacBio long reads sequencing platform must be introduced for the whole genome sequencing of shrimp.

Taking advantage of massively parallel sequencing technology, a total of 114,829 SLAF-tags with 100 bp length were generated in this study. Considering the total genome size was estimated as being approximately 2.6 Gb, the SLAF-tag sequences accounted for approximately 0.44% of the total genome sequence. Among these SLAF-tags, 25,140 polymorphic markers were discovered (Table 3). The major advantage of SNP over AFLP and RAPD markers is their transferability between different linkage maps and labs. Because each marker developed in this study contained a 100-bp genome sequence, comparative genomic analysis is possible and it might be helpful in genome assembly.

Based on the sequencing data, the mutation (SNP and indel) frequency was analyzed and a mutation ratio of 9 mutations per Kb was observed. This ratio is lower than that of eastern oyster (1 per 20 bp)^38^, however it is higher than most of the reported species, including common carp (4.87 per 1 Kb)^30^, Sunflowers (7 per 1 Kb)^39^, watermelon (7.14 per 1 Kb)^40^ and humans (3.3 per 1 Kb)^41^. Because the mutation frequency was generated from only two samples (parents of the mapping family), the actual mutation frequency for the population could be higher.

In a previous report, the genotyping error rate decreased greatly as read depth increased. When the read depth increased to 12, the error rate could almost be ignored^30^. For the raw data, the read depths of all developed makers ranged from 7.79-fold to 24.29-fold. However, the data were filtered to exclude low read depth markers prior to mapping, so the average read depth of markers included in the linkage map was as high as 27-fold. This read depth was much higher than that previously reported. The great read depth of these markers ensured a high accuracy of marker genotyping.

The linkage map constructed here contained 44 linkage groups, which is consistent with karyotypes of *L. vannamei*^42^. In previously reported linkage maps constructed by AFLP and SSR markers, more than 44 linkage groups were observed. The extra linkage groups observed in these studies may have been caused by the limited intermediate markers that linked groups belonging to the same chromosome together. In this study, a total of 6,146 markers were mapped in 44 sex-averaged linkage groups. This large number of markers and their even distribution facilitated the full-scale map coverage.

Because more than 6,000 markers were genotyped in 205 offspring, the limited meiotic events resulted in several markers clustering together at one position. These clustered markers were referred as “bin signatures”. A bin signature comprises the consensus segregation pattern of marker loci that do not have any recombination and thus a marker interval of zero^40^. In the linkage map, a total of 1,031 (male map), 1,181 (female map) and 1,630 (sex-averaged map) markers were observed in “bin signatures”. In the “bin signatures”, markers were known to be clustered together but the orientation was unknown, which may influence the scaffold orientation in the genome assembly. In future studies, more families and offspring need to be genotyped to separate the markers in the “bin signatures”.

Using the SLAF-seq approach, only regions near the enzyme sites are sequenced. The uneven distribution of enzyme sites resulted in the uneven distribution of markers along the linkage map. Moreover, the sequence from SLAF-seq covered 0.44% of the total genome; the low coverage might be another limiting factor affecting marker distribution. Even so, >90% of marker interval spaces were <5 cM. The regions with large marker intervals may be a result of the large number of repeat sequences in the *L. vannamei* genome. In this study, the average intermarker distance was 0.7 cM in the sex-average map, which is much lower than that in previous linkage maps^12^,^13^,^22^,^23^,^24^. To our knowledge, this is the linkage map with the highest density for *L. vannamei* to date. Compared to the other linkage maps constructed using next-generation sequencing technology for aquaculture species^28^,^29^,^30^, the intermarker distance was also shorter.

In penaeids, conflicting results of sex difference in recombination were observed in different studies. For example, the linkage map of *P. monodon* constructed by Wilson *et al*. showed slightly lower recombination rates in males than females^43^. However, in the *P. monodon* linkage map constructed by Staelens *et al*., male and female recombination frequencies did not differ significantly^44^. For *L. vannamei*, conflicting results were also reported between the AFLP linkage map and the gene-based SNP linkage map^13^,^22^. These conflicting results may have been derived from the marker types and marker density. The previous linkage maps were constructed mainly using co-dominant AFLP markers. A lack of common markers between male and female maps could have influenced the accurate estimation of male to female recombination ratios. In this study, we had large numbers of common markers between male and female maps, thus the estimated recombination frequency between the sexes should be more accurate.

Integration of a high-resolution genetic linkage map with reference genomic scaffolds and BAC clones will be helpful for the improvement of genome assembly by orienting the genomic scaffolds. In the sex-averaged linkage map, a total of 5,044 markers were anchored to 4,908 scaffolds. These 4,908 scaffolds were mapped onto chromosomes, and the relative distance between them was known. The large number of anchored markers (82.07%) will be useful for further genome assembly.

The genome survey mitigated for the lack of sequence information generated from the SLAF-seq platform. The SLAF-seq platform only generated markers with 100-bp sequence, which is short for further use in genetic studies. However, after blasting the marker sequence to the assembled preliminary genome sequence, the markers could be matched to sequences of 38 Kb or more. In addition to the scaffolds, some BAC clones were anchored to the linkage map, which may help further fine mapping of interesting parts of the genome. Above all, from the marker integration, we can obtain a preliminary arrangement of sequences along chromosomes of *L. vannamei*. The integration also verified the accuracy of the constructed linkage map. Some markers that grouped together were anchored to the same scaffold with the same orientation. For example, in LG 1, Marker51641 and Marker13241 were near each other and both were linked to scaffold434749. This result partially validated the high accuracy of the linkage map.

One of the principal applications of the genetic linkage map was QTL mapping of interesting traits. QTL mapping of growth traits has been conducted in fish^45^,^46^, shrimp^24^,^47^, bivalve mollusks^48^ and many other aquaculture species. Many QTLs related to growth traits have been reported, among which some had a high resolution and some did not. For *L. vannamei*, QTL mapping was conducted based a linkage map constructed with 429 AFLPs and 22 SSRs markers with an average marker space of 7.6 cM. Considering the lower marker density and the limitation of the transferability of AFLP markers among different families, it is difficult to identify growth related markers for marker assisted breeding. Here, QTLs for body weight and body length were analyzed based on a high-density linkage map, which allowed for greater resolution of QTL locations. As a result, several markers were determined to be directly linked with the growth traits. These markers were located in different linkage groups, which reflected the complexity of these polygenic traits. For body weight and body length, the LOD curve of the QTL analysis (Figure S7) indicated that the two traits may be controlled by similar genes. However, as the threshold estimated by a permutation test was relatively high, the markers that were significant for both body weight and body length were relative few. Only one marker (Marker7605) was found to be significant for both traits.

## Conclusions

In this study, a genome survey was conducted for *L. vannamei* and a preliminary reference genome was assembled. A high percentage of repetitive sequences and possibly high genome heterozygosity were observed in this species. Based on the reference genome, a high-density genetic linkage map was constructed using the SLAF-seq method. The linkage map contained 44 linkage groups with a low intermarker distance. This high-density linkage map serves as a foundation of genetic knowledge for *L. vannamei*. QTLs for body length and body weight were identified and will be useful in marker-assisted selection studies for this important aquaculture species. These genomic resources may also play an important role in future whole genome sequencing projects and genetic breeding studies in penaeid shrimp.

## Materials and Methods

### Genome survey sequencing and analysis

The DNA of *L. vannamei* was extracted from muscle for sequencing. Three paired-end DNA libraries with a gradient insert size of 170 bp, 300 bp and 500 bp were constructed following the standard Illumina operating procedure. To elongate scaffolds, two mate-paired libraries with an insert size of 1 Kb and 2 Kb were also constructed. Both paired-end and mate-paired sequencing was performed on the Illumina platform (Illumina, Inc.; San Diego, CA, USA).

All the raw data were trimmed to filter out low-quality data and adapter contaminates with the help of NGS QC Toolkit^49^. A *de novo* assembly procedure was performed on the clean reads to construct contigs using SOAPdenovo software (http://soap.genomics.org.cn/soapdenovo.html) with the following parameters: the k value in K-mer was set at 45, unsolve repeats by reads and fill gaps in scaffolds. After that, mate-paired clean reads were added to link the contigs into scaffolds.

### Mapping family preparation and DNA extraction

The full-sib family for linkage map construction was created in the breeding center of Guangxi Institute of Fisheries. First, four candidate families were created. The parents of the four candidate families were artificially inseminated and their progeny were cultured in different ponds. The genetic distances between the four parents were determined using 10 previously reported microsatellite loci^24^. The family showing the largest genetic distance was selected as the mapping family. A total of 205 progenies were randomly selected and body weight and length were measured for each individual.

Genomic DNA of parents and progeny were extracted using a TIANGEN Marine animal DNA extraction kit (TIANGEN, Beijing, China). The concentration of extracted DNA was determined using a NanoDrop 1000 Spectrophotometer (NanoDrop, Wilmington, DE, USA). DNA integrity of each individual was evaluated by gel electrophoresis.

### In-silico analysis of restriction enzyme recognition sites

Based on the assembled primary reference genome from the genome survey, we performed in-silico analysis of the 30 common restriction enzyme sites in the reference genome using Perl script^27^. The distribution of digestion sites, the total number of digestion sites and the lengths of the resultant fragments were investigated. The best enzyme combination was chosen based on the expected fragment number, genome-wide distributed of digestion sites and low number of repeated sequence.

### SLAF library construction and sequencing

The SLAF library was constructed as described previously^30^. Based on the in-silico analysis, two enzymes, endonuclease *Eco*R I and *Nla* III, were used to digest the genome. In brief, genomic DNA from each individual was digested with restriction endonuclease *Eco*R I (New England Biolabs [NEB], Ipswich, MA, USA) and ligated to an *Eco*R I adapter. Then, the DNA was digested with an additional restriction enzyme, *Nla* III. PCR reactions were used to amplify the digested DNA and barcodes were added to the different samples. The PCR products were purified using an E.Z.N.A.H Cycle Pure Kit (Omega, Norcross, GA, USA) and samples with different barcodes were pooled together. A total of 4 DNA pools were constructed, each of which included the two parents and approximately 50 progeny. After adding the Solexa adapter to the DNA fragment, the pooled DNA was purified using a Quick Spin column (Qiagen, Hilden, Germany) and run out on agarose gel. DNA fragments, which included adapters and barcodes, from 500 to 550 were separated and purified using a Gel Extraction Kit (Qiagen). Then, DNA fragments were amplified using Phusion Master Mix (NEB) and Solexa Amplification primer^27^. PCR products were purified using the QIAquick PCR Purification Kit (Qiagen) and then diluted for sequencing. All the experiments were accomplished at Beijing Biomarker Technologies Co. Ltd. The four libraries obtained in this way were sequenced using the paired-end sequencing method on an Illumina HiSeq 2500 platform (Illumina, Inc., San Diego, CA, USA).

### Marker development and genotyping

Marker genotyping was similar to that described previously, with some modifications^26^,^27^. Raw reads were separated by barcodes, and reads with quality scores below 20 were discarded. Barcodes were trimmed from reads, and the reads was truncated to a length of 50 base sequences at each end. Due to the lack of a shrimp reference sequence, a Perl script was written to group the SLAF paired-end reads with clear index into SLAF loci based on sequence similarity. To reduce computational demands, identical reads were amalgamated, and sequence similarity was analyzed using one-to-one alignment by BLAT^15^. SLAF loci with more than 4 tags were filtered out because a SLAF locus can contain no more than 4 genotypes in mapping populations of diploid species. SLAF tags with sequence errors were corrected to the most similar genotype.

### Linkage map construction

The linkage map was constructed using HighMap, as described by Liu *et al*.^50^. The HighMap software used an iterative ordering and error correction strategy to construct the high-density genetic maps. Low-quality markers, such as those with low depth or those lacking individual information, were filtered out. Then, high-quality markers were imported into HighMap, and the Mendel segregation ratio of markers was checked by the chi-square test (P < 0.05)^51^. Linkage groups were determined using a pair-wise modified independence test LOD score (logarithm of odds) in the grouping module of HighMap^50^. The enhanced algorithm of Gibbs sampling, spatial sampling and simulated annealing (GSS) was employed to order markers in the marker ordering module (see Liu *et al*. 2014 for detailed methods). To reduce computational load, the GSS was enhanced using the summation of adjacent recombination fractions (SARF) and by adopting a Blocked Gibbs sampler in simulated annealing. During the iterative marker ordering process, the error correction strategy of SMOOTH and a k-nearest neighbor algorithm^52^ was used to correct genotyping errors and impute missing genotypes. After three cycles of iterative marker ordering and error correction, an optimal map of sampled markers was obtained. Recombination values were converted to genetic distances in centiMorgans (cM) based on the Kosambi mapping function. The integrated map was computed using the Combine Group for Map Integration function. A genetic linkage map was drawn using MapChart ver. 2.2^53^.

### Genome size and coverage estimation

The estimated genome size (*Ge*) was calculated using two methods. The average marker spacing (*s*) in each linkage group was calculated by dividing the total length of each linkage group by the number of intervals. Genome estimation size 1 (*Ge1*) was calculated by adding 2 *s* to the length of each linkage group^54^. Genome estimation size 2 (*Ge2*) was determined by taking the total length of the linkage groups multiplied by the factor (m + 1)/(m − 1), where m is the number of loci on each linkage group^55^. The final estimated genome size for *L. vannamei* was taken as the average of the two estimates. The map coverage was calculated by dividing the estimated genome size by the summed length of all linkage groups.

### Integration of linkage map with genomic scaffolds and BAC library

Integration analysis was performed between the linkage map and genomic scaffolds using BLASTn. If both ends of a marker matched the same scaffolds with the highest BLAST score, the marker and the corresponding scaffold were integrated. If both ends of a marker showed homologies to different scaffolds, then the marker was linked to the two scaffolds. In previous work by our group, a BAC library was constructed for *L. vannamei*^1^, and a total of 4,609 paired-end BAC-end sequences (BESs) were obtained by Sanger sequencing^2^. To integrate genomic scaffolds with the sequenced BAC clones, the scaffolds were blasted against the BESs using BLASTn with an E-value cutoff of 1E-10.

### QTL mapping of growth traits

The phenotypic data are given in Supplementary Table S5. The distributions of body weight and body length were assessed using Shapiro-Wilk Normality Test implemented in R software. Because the “bin signature” may influenced detection power due to high LD among markers, we selected one marker from each “bin signature” to represent the “bin signature” group. As a result, a total of 4626 representative markers were used for further QTL mapping analysis. QTL analysis was carried out using the Composite Interval Mapping method in the program Windows QTL Cartographer V2.5^56^. The CIM analysis was run using Model 6 with four parameters for forward and backward stepwise regression, a 10-cM window size, five control markers, and a 1-cM step size. The LOD score significance thresholds were calculated using permutation tests with an experiment-wise significance level of 0.05, n = 1000. A QTL was determined to be significant if the LOD score was higher than the significance threshold estimated by permutation. The Multiple Interval Mapping (MIM) method was also used to re-estimate the QTL effect and to more precisely locate the QTLs. The additive effect and percentage of phenotypic variation explained by each QTL (R^2^) were obtained from the final CIM results. The total genetic variance explained by all QTL (total R^2^) was estimated by MIM in Windows QTL Cartographer V2.5^57^,^58^,^59^.

## Additional Information

**How to cite this article**: Yu, Y. *et al*. Genome survey and high-density genetic map construction provide genomic and genetic resources for the Pacific White Shrimp *Litopenaeus vannamei*. *Sci. Rep*. **5**, 15612; doi: 10.1038/srep15612 (2015).

## Supplementary Material

Supplementary Figures

Supplementary Data S1

Supplementary Table S1

Supplementary Table S2

Supplementary Table S3

Supplementary Table S4

Supplementary Table S6

## Figures and Tables

**Table 1:** Summary of genome sequencing data of *L. vannamei*.

Insert Size (bp)	Reads Length (bp)	Total Data (Gb)	Sequencing Coverage (X)
170	100	13.09	5.34
300	100	27.81	11.36
500	100	49.78	20.32
1000	80	7.05	2.71
2000	80	11.12	4.28
Total	—	108.85	41.86

**Table 2:** Statistics of genome assembly of *L. vannamei*.

	Contig	Scaffold
Number	6,908,022	4,336,336
Total length (bp)	2,306,928,471	2,371,197,647
>2Kb	30,791	252,373
Longest (bp)	21,769	38,588
Shortest (bp)	100	100
Mean Length (bp)	334	547
N50 (bp)	409	1,343
N90 (bp)	152	180

**Table 3:** Statistics of developed SLAF markers.

Type	SLAF number	Total depth
Polymorphic SLAF	25,140	66,918,826
Non-polymorphic SLAF	89,689	117,986,668
Total	114,829	184,905,494

**Table 4:** Statistics of 44 linkage groups in constructed sex-averaged map, male map, and female map.

LG ID	Sex-averaged map			female map			male map		
	Number of markers	Distance (cM)	Average distance (cM)	Number of Markers	Distance (cM)	Average distance (cM)	Number of markers	Distance (cM)	Average Distance (cM)
LG1	279	166.14	0.6	204	154.12	0.76	182	157.343	0.87
LG2	84	70.64	0.85	63	166.09	2.68	58	148.54	2.61
LG3	171	88.06	0.52	114	86.44	0.76	131	96.046	0.74
LG4	101	112.58	1.13	59	109.12	1.88	61	115.875	1.93
LG5	99	69.02	0.7	74	292.51	4.01	67	157.764	2.39
LG6	105	113.83	1.09	75	221.17	2.99	85	114.373	1.36
LG7	104	81.72	0.79	71	52.57	0.75	71	256.945	3.67
LG8	79	131.56	1.69	56	113.84	2.07	58	122.903	2.16
LG9	194	90.15	0.47	141	90.67	0.65	123	73.636	0.6
LG10	143	87.18	0.61	99	205.34	2.1	90	213.16	2.4
LG11	110	65.07	0.6	59	60.55	1.04	74	192.228	2.63
LG12	92	83.64	0.92	60	90.48	1.53	68	172.738	2.58
LG13	186	94.28	0.51	141	95.34	0.68	119	99.436	0.84
LG14	21	21.36	1.07	11	33.19	3.32	16	36.414	2.43
LG15	201	76.22	0.38	125	228.79	1.85	160	76.886	0.48
LG16	169	101.8	0.61	123	110.06	0.9	135	336.987	2.51
LG17	189	100.68	0.54	137	93.79	0.69	119	111.767	0.95
LG18	117	98.52	0.85	70	106.3	1.54	89	100.092	1.14
LG19	183	92.41	0.51	132	254.25	1.94	122	99.298	0.82
LG20	226	117.02	0.52	190	363.78	1.92	132	125.893	0.96
LG21	26	33.32	1.33	19	35.24	1.96	16	16.573	1.1
LG22	113	83.55	0.75	83	145.66	1.78	76	87.21	1.16
LG23	52	78.37	1.54	42	82.71	2.02	17	55.087	3.44
LG24	99	95.06	0.97	71	288.3	4.12	73	96.322	1.34
LG25	178	95.63	0.54	152	97.92	0.65	68	103.642	1.55
LG26	126	106.17	0.85	100	108.77	1.1	92	106.995	1.18
LG27	131	102.13	0.79	98	101.34	1.04	93	106.41	1.16
LG28	181	110.12	0.61	112	120.29	1.08	137	106.646	0.78
LG29	222	124.16	0.56	140	127.26	0.92	155	145.603	0.95
LG30	100	103.29	1.04	71	101.87	1.46	82	375.004	4.63
LG31	175	113.19	0.65	138	102.32	0.75	97	117.718	1.23
LG32	172	101.38	0.59	140	94.15	0.68	96	316.267	3.33
LG33	91	48.14	0.53	73	94.29	1.31	44	396.348	9.22
LG34	222	120.05	0.54	165	126.67	0.77	155	113.405	0.74
LG35	107	108.42	1.02	80	113.55	1.44	73	105.397	1.46
LG36	153	108.05	0.71	99	121.57	1.24	115	116.648	1.02
LG37	154	99.89	0.65	122	100.52	0.83	105	104.039	1
LG38	96	119.54	1.26	55	115.94	2.15	58	107.007	1.88
LG39	75	131.67	1.78	53	124.25	2.39	64	132.007	2.1
LG40	156	130.85	0.84	106	112.62	1.07	123	131.694	1.08
LG41	110	82.65	0.76	82	85.24	1.05	78	174.613	2.27
LG42	189	111.24	0.59	132	124.32	0.95	153	114.689	0.75
LG43	178	102.01	0.58	127	107.91	0.86	126	119.415	0.96
LG44	187	100.67	0.54	132	96.34	0.74	145	86.887	0.6
Total	6,146	4,271.43	0.7	4,396	5,657.42	1.29	4,201	6,143.95	1.46

**Table 5:** Mendelian segregation analysis results using Chi-square test.

P-value	Marker Number	Percent
P ≥ 0.01	5,352	87.08%
0.05 < p < 0.01	388	6.31%
P ≤ 0.05	406	6.61%
Total	6,146	100.00%

**Table 6:** Recombination rates in male and female maps using shared markers.

LG ID	Female	Male	Female/male ratio	LG ID	Female	Male	Female/male ratio
LG1	153.122	139.875	1.09	LG23	63.769	55.087	1.16
LG2	166.089	148.54	1.12	LG24	288.297	95.672	3.01
LG3	86.442	92.825	0.93	LG25	97.408	103.567	0.94
LG4	108.119	114.875	0.94	LG26	108.767	106.995	1.02
LG5	292.506	157.764	1.85	LG27	96.265	106.41	0.9
LG6	220.166	113.195	1.95	LG28	119.618	105.608	1.13
LG7	47.658	209.522	0.23	LG29	126.258	144.603	0.87
LG8	112.843	110.65	1.02	LG30	101.87	375.004	0.27
LG9	87.076	72.636	1.2	LG31	102.318	111.585	0.92
LG10	204.851	203.341	1.01	LG32	81.642	294.864	0.28
LG11	60.551	140.105	0.43	LG33	94.287	396.348	0.24
LG12	89.658	172.738	0.52	LG34	123.393	112.405	1.1
LG13	94.951	99.436	0.95	LG35	113.549	105.174	1.08
LG14	22.567	13.152	1.72	LG36	121.569	114.299	1.06
LG15	227.806	70.959	3.21	LG37	99.521	103.039	0.97
LG16	110.059	336.494	0.33	LG38	114.941	106.007	1.08
LG17	93.792	107.972	0.87	LG39	124.251	124.929	0.99
LG18	106.297	99.644	1.07	LG40	112.619	111.094	1.01
LG19	254.248	99.181	2.56	LG41	77.813	174.613	0.45
LG20	363.78	125.754	2.89	LG42	124.316	114.689	1.08
LG21	34.239	15.573	2.2	LG43	107.91	119.415	0.9
LG22	145.661	75.748	1.92	LG44	95.339	84.351	1.13
				Total	5578.20	5885.74	0.94

**Table 7:** Detected QTLs for Body Length.

LG	Position (cM)	Markers	LOD	Additive Effect	Dominant Effect	R^2^ (%)
1	51.3	Marker12047	6.1	−18.4	16.5	13.1
10	39.3	Marker7605	5.7	−10.4	12.1	19.0
12	50.3	Marker2797	5.3	−13.9	15.4	14.7
13	67.3	Marker18522	5.5	−14.7	2.9	15.0
18	66.0	Marker33221	5.5	−9.2	7.7	22.6
33	25.9	Marker24250	6.5	−16.3	20.3	17.9
37	35.6	Marker8838	5.1	−17.7	16.5	11.3
38	53.4	Marker11898	5.9	−15.7	20.2	17.9
38	64.6	Marker42558	5.4	−16.5	19.2	13.8
42	44.9	Marker89109	5.8	−11.7	12.9	16.3
44	17.6	Marker5684	5.1	9.5	11.7	19.7

**Table 8:** Detected QTLs for Body Weight.

LG	Position (cM)	Locus	LOD	Additive Effect	Dominant Effect	R^2^ (%)
9	46	Marker34000	7.1	−3.6	2.3	20.8
10	39.3	Marker7605	4.1	−4.0	5.1	14.3
22	28.8	Marker33688	4.2	1.1	1.0	7.8
27	13.5	Marker21173	3.8	−4.0	3.1	15.5
35	61.1	Marker58445	4.0	−2.2	0.2	13.8
38	38.7	Marker4670	3.7	−1.4	0.5	6.4
41	38.5	Marker10074	4.1	−5.2	4.7	11.1
