# Supplementary material for: Genome survey and high-density genetic map construction provide genomic and genetic resources for the Pacific White Shrimp Litopenaeus vannamei
Source: Sci Rep. 2015 Oct 27;5:15612. doi: 10.1038/srep15612 (PMC4621519; doi:10.1038/srep15612)
Supplement: Supplementary Figures [file srep15612-s1.pdf]

Genome survey and high-density genetic map construction provide genomic and genetic resources for Pacific White Shrimp *Litopenaeus vannamei*

Yang Yu[1†], Xiaojun Zhang[1†], Jianbo Yuan[1], Fuhua Li[1] , Xiaohan Chen[2],Yongzhen Zhao[2], Long Huang[3], Hongkun Zheng[3*], Jianhai Xiang[1*]

[1] Key Laboratory of Experimental Marine Biology, Institute of Oceanology, Chinese Academy of Sciences, Qingdao 266071, China.

[2] Guangxi Key Laboratory of Aquatic Genetic Breeding and Healthy Aquaculture, Guangxi Academy of Fishery Sciences, Nanning 530021, China.

[3] Biomarker Technologies Corporation, Beijing 101300, China.

† These authors contributed equally to this work.

* Corresponding author:

Jianhai Xiang, Institute of Oceanology, Chinese Academy of Sciences, Qingdao, China. Fax: +86-0532-82898568. E-mail addresses: jhxiang@qdio.ac.cn;

Hongkun Zheng, Biomarker Technologies Corporation, Beijing 101300, China. Fax: +86-010-57045001. E-mail addresses: zhenghk@biomarker.com.cn.

Excel Spreadsheets of Supplementary Data S1 and Supplementary Table S1-S5, available as separate files.

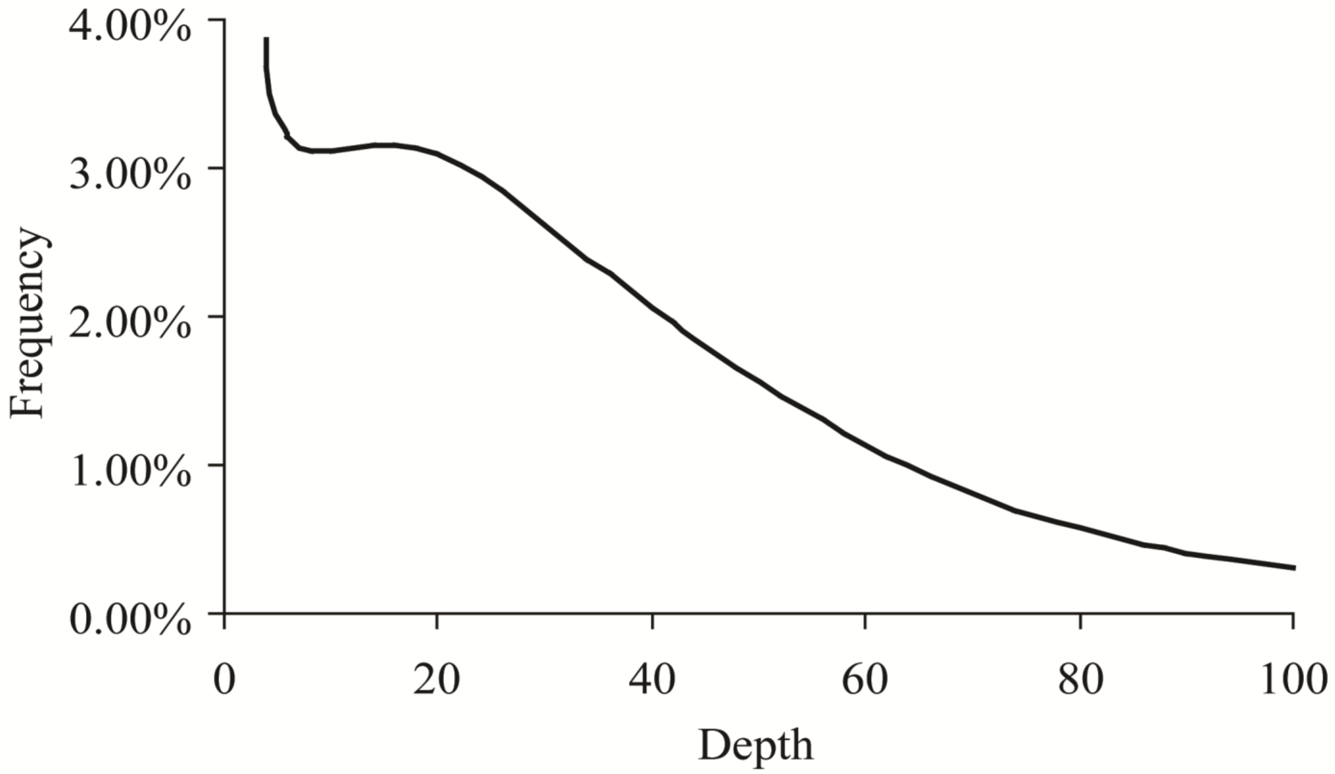

Figure S1: K-mer curve of *L. vannamei* in genome survey analysis.

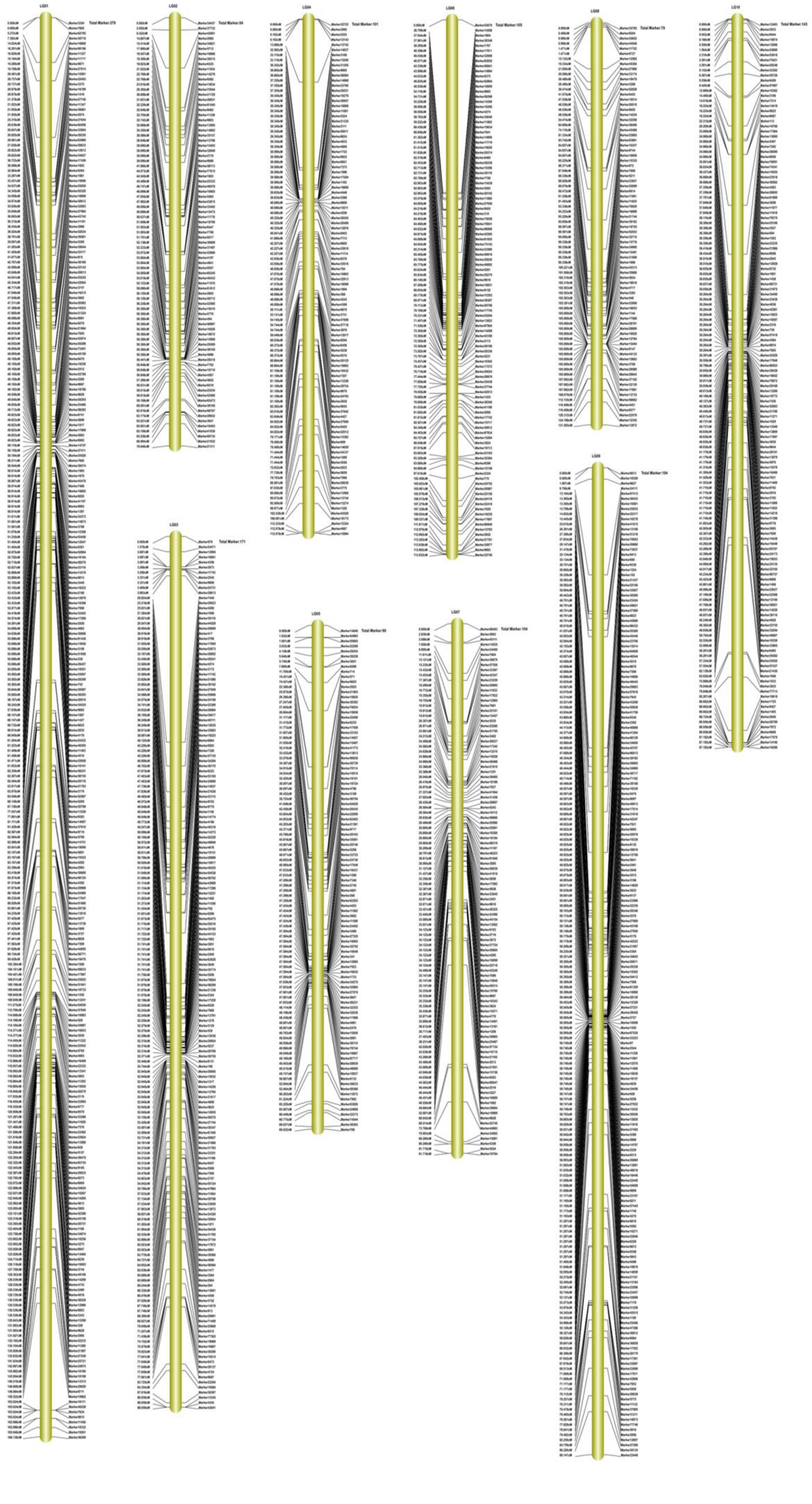

Figure S2: Sex-averaged genetic linkage group 1-10 of *L.vannamei*.

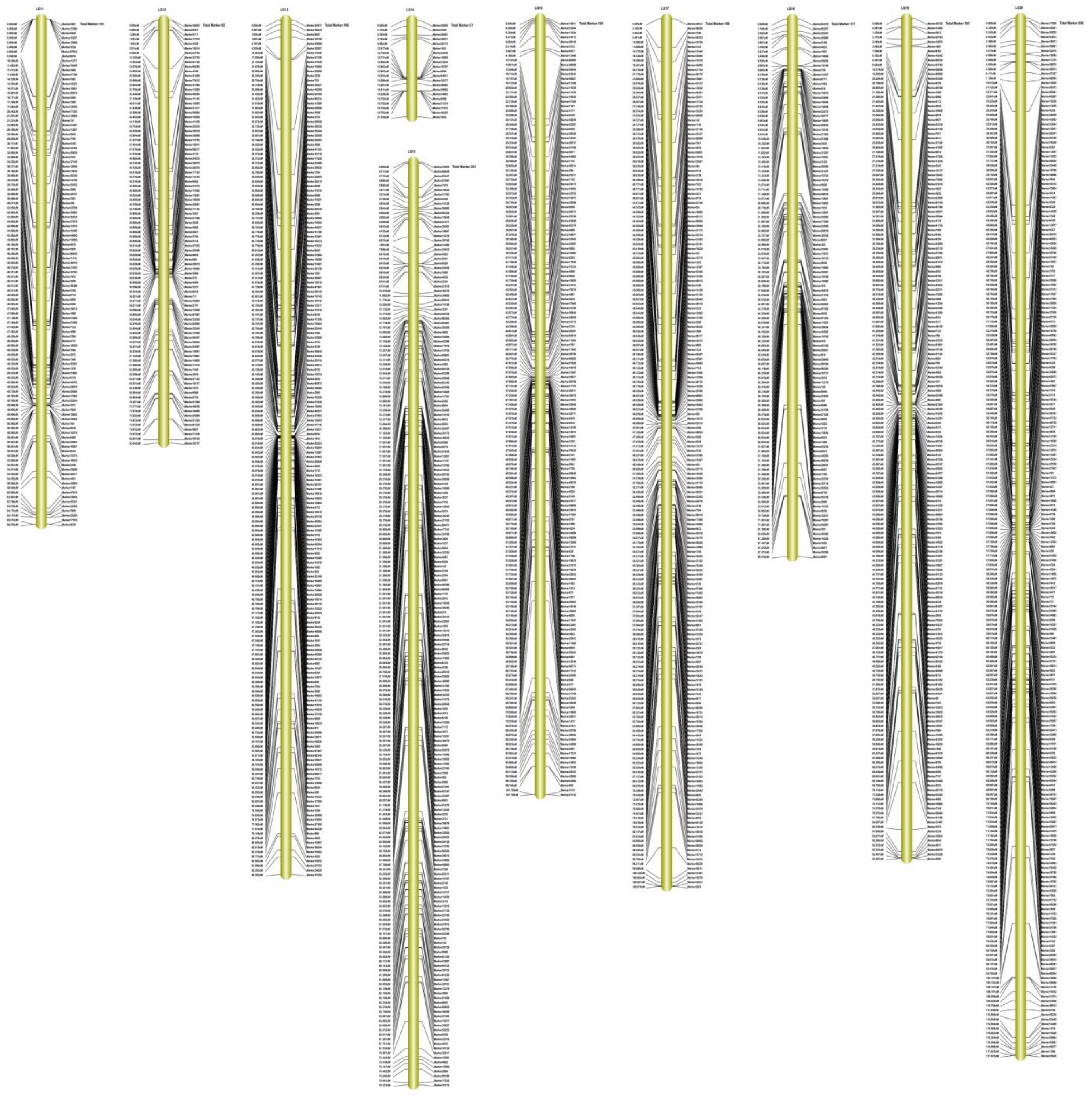

Figure S3: Sex-averaged genetic linkage group 11-20 of *L.vannamei*.

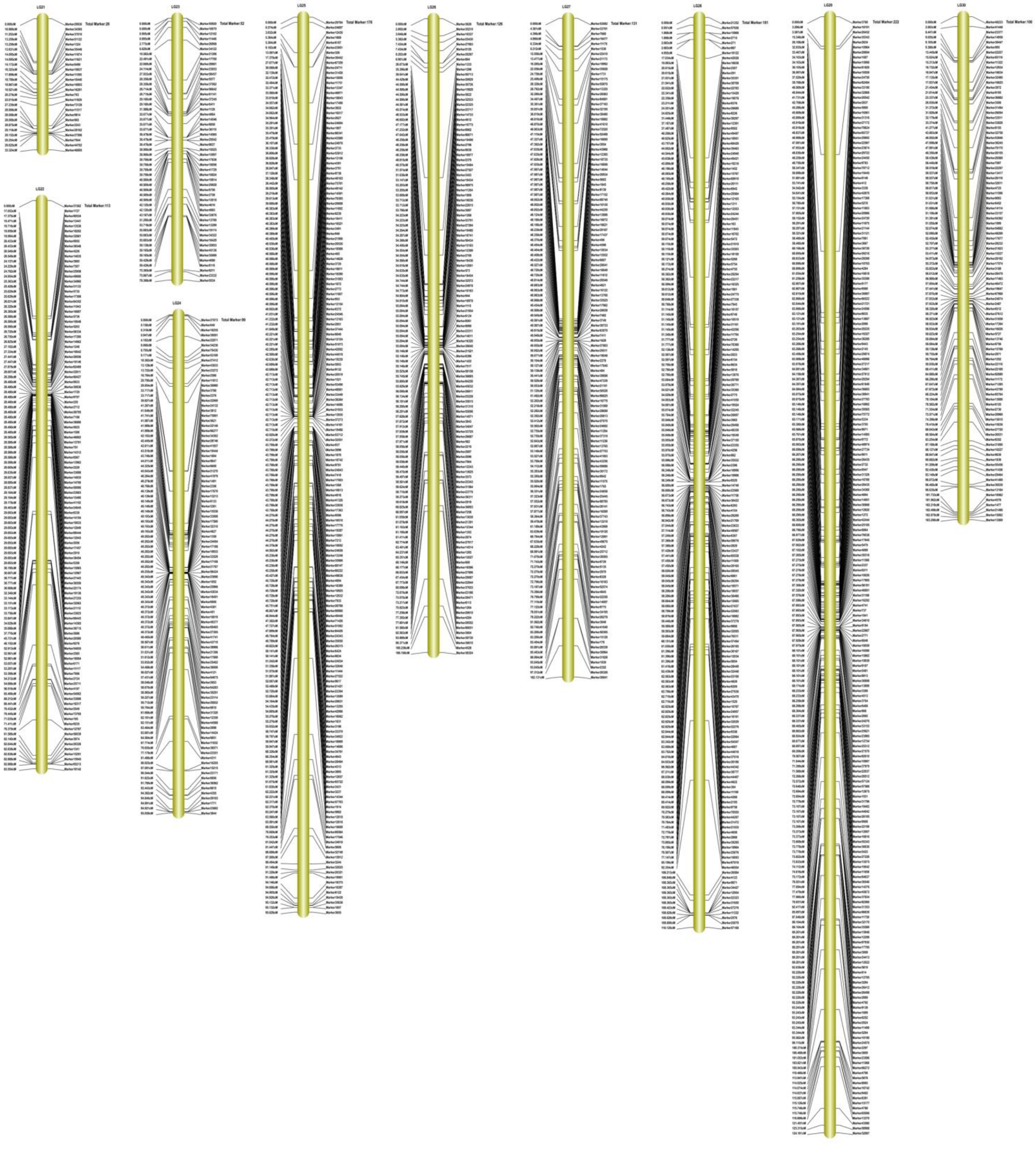

Figure S4: Sex-averaged genetic linkage group 21-30 of *L.vannamei*.

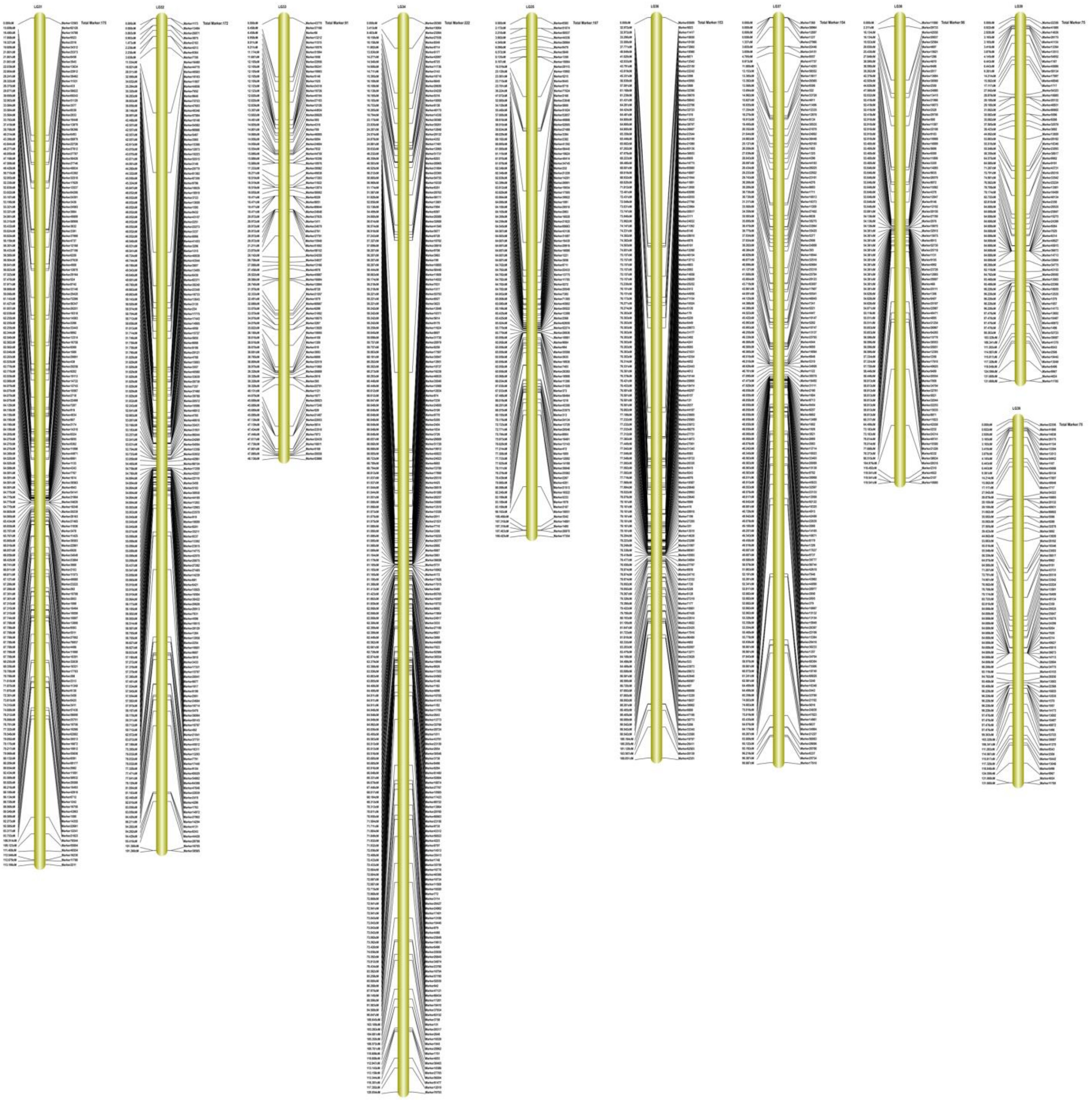

Figure S5: Sex-averaged genetic linkage group 31-40 of *L.vannamei*.

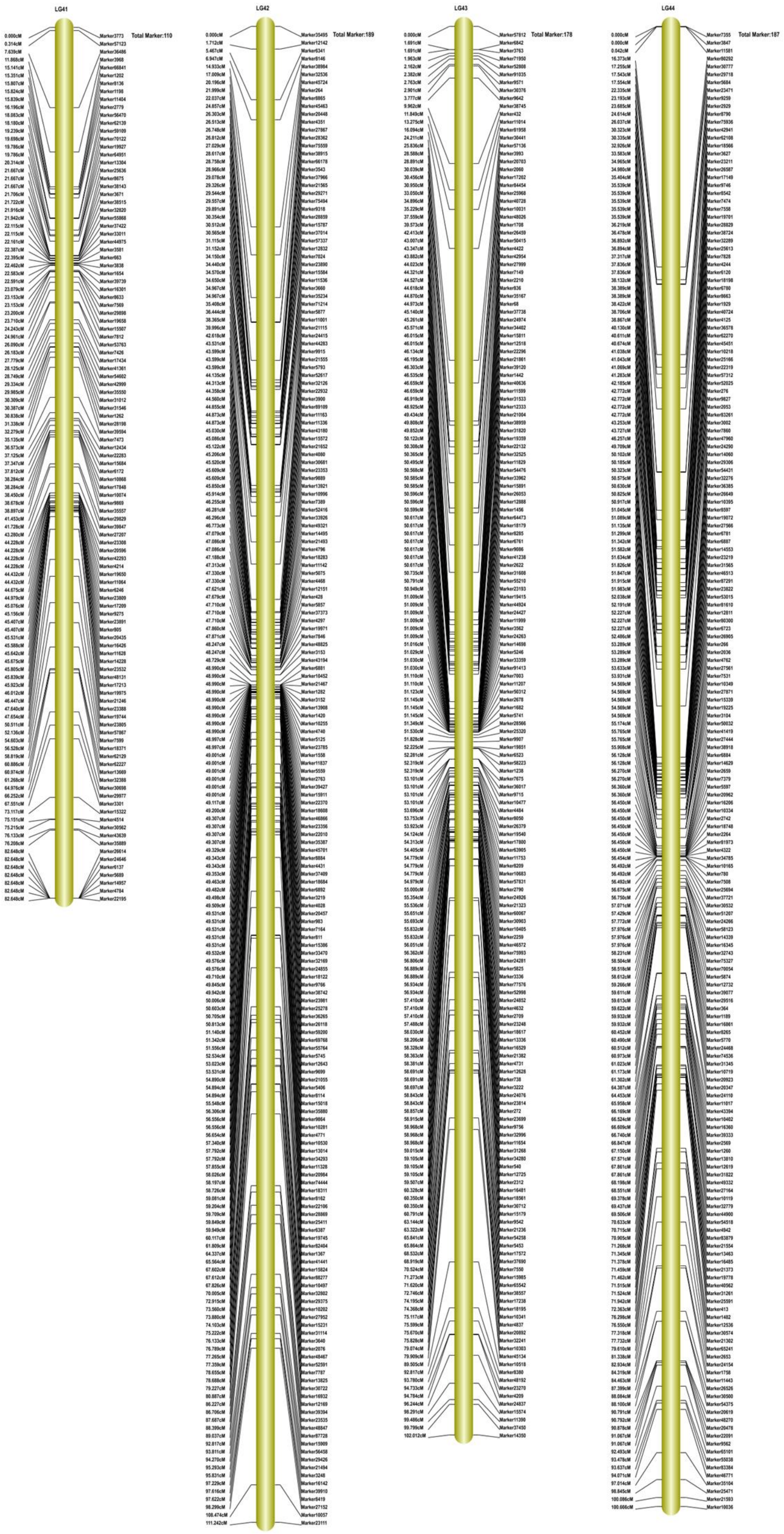

Figure S6: Sex-averaged genetic linkage group 41-44 of *L.vannamei*.

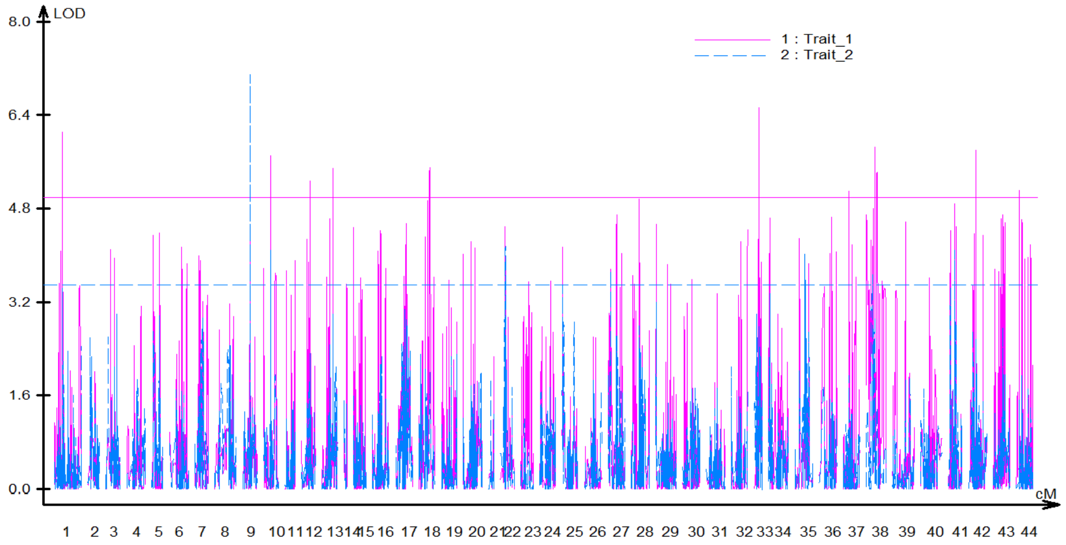

Figure S7: QTLs mapping result of body length (Trait_1) and body weight (Trait_2) by CIM method in Windows QTL Cartigrapher.
